# Human papillomavirus dysregulates the cellular apparatus controlling the methylation status of H3K27 in different human cancers to consistently alter gene expression regardless of tissue of origin

**DOI:** 10.18632/oncotarget.19885

**Published:** 2017-08-03

**Authors:** Steven F. Gameiro, Bart Kolendowski, Ali Zhang, John W. Barrett, Anthony C. Nichols, Joe Torchia, Joe S. Mymryk

**Affiliations:** ^1^ Department of Microbiology and Immunology, The University of Western Ontario, London, ON N6A 3K7, Canada; ^2^ Department of Biochemistry, The University of Western Ontario, London, ON N6A 3K7, Canada; ^3^ Department of Otolaryngology, Head & Neck Surgery, The University of Western Ontario, London, ON N6A 3K7, Canada; ^4^ Department of Oncology, The University of Western Ontario, London, ON N6A 3K7, Canada; ^5^ London Regional Cancer Program, Lawson Health Research Institute, London, ON N6C 2R5, Canada

**Keywords:** cancer, human papillomavirus, epigenetics, gene expression, methylation

## Abstract

High-risk human papillomaviruses (HPV) cause cancer at multiple distinct anatomical locations. Regardless of the tissue of origin, most HPV positive (HPV+) cancers show highly upregulated expression of the p16 product of the cyclin-dependent kinase inhibitor 2A (CDKN2A) gene. Paradoxically, HPV+ tumor cells require continuous expression of this tumor suppressor for survival. Thus, restoration of normal p16 regulation has potential therapeutic value against HPV induced cancers. Normally, p16 transcription is tightly controlled at the epigenetic level via polycomb repressive complex-mediated tri-methylation of histone 3 lysine 27 (H3K27me3). Although a mechanism by which HPV induces p16 has been proposed based on tissue culture models, it has not been extensively validated in human tumors. In this study, we used data from over 800 human cervical and head and neck tumors from The Cancer Genome Atlas (TCGA) to test this model. We determined the impact of HPV status on expression from the CDKN2A locus, the adjacent CDKN2B locus, and transcript levels of key epigenetic regulators of these loci. As expected, HPV+ tumors from both anatomical sites exhibited high levels of p16. Furthermore, HPV+ tumors expressed higher levels of KDM6A, which demethylates H3K27me3. CpG methylation of the CDKN2A locus was also consistently altered in HPV+ tumors. This data validates previous tissue culture studies and identifies remarkable similarities between the effects of HPV on gene expression and DNA methylation in both cervical and oral tumors in large human cohorts. Furthermore, these results support a model whereby HPV-mediated dysregulation of CDKN2A transcription requires KDM6A, a potentially druggable target.

## INTRODUCTION

Human papillomaviruses (HPV) that infect mucosal tissues are classified as low- or high-risk based on the frequency with which they are associated with cancer. Although both types dysregulate normal cell growth, infection by high-risk HPV is a causative agent for cancers at multiple distinct anatomical subsites [1, 2]. HPV was first recognized as the cause of virtually all cervical carcinomas, and was then subsequently found to contribute to other anogenital cancers as well [2–5]. More recently, HPV infection was recognized as the agent responsible for an epidemic of oropharyngeal cancers [6, 7]. HPV has also been suggested to cause a subset of lung, prostate, bladder, and breast cancers [8–11]. Taken together, HPV infection is thought to be responsible for at least 5% of human cancers worldwide [12].

HPV encodes two main oncogenes, E6 and E7 [13]. Both are constitutively expressed in HPV positive (HPV+) tumors, and suppression of expression of either of these two viral proteins causes HPV dependent tumor cells to senesce and die [1, 14–19]. Both E6 and E7 perform multiple functions in an infected and/or cancerous cell. These two viral oncoproteins function by interacting with multiple key cellular regulatory proteins to dysregulate gene expression and growth [13]. For example, p53 is bound and degraded by E6, while the retinoblastoma protein (Rb) and family members are important targets of E7 [20, 21]. Rb controls exit from the G1 phase of the cell cycle, and the interaction and subsequent degradation of Rb by E7 induces inappropriate cell cycle progression in HPV infected cells [22, 23]. In high-risk HPV infections, viral oncoproteins function to uncouple cell growth and differentiation and contribute to the formation of epithelial dysplasia, which may progress to carcinoma if the infection is not resolved [13].

The HPV E7 oncoproteins alter the regulation of many host cellular genes and much effort has been devoted to understanding the mechanisms by which this occurs [24]. A subset of the effects of E7 on the cellular transcriptome are mediated by epigenetic changes [25]. Specifically, E7 expression has been reported to reduce the global levels of tri-methylated lysine 27 on histone 3 (H3K27me3), a mark of transcriptionally silenced chromatin [26–28]. Paradoxically, several cell culture studies have shown that HPV induces a significant upregulation in the expression level of the enhancer of zeste homolog 2 (EZH2) component of the polycomb repressive complex 2 (PRC2) [28–30], which is the methyltransferase responsible for mono-, di-, and tri-methylation of H3K27 [27, 31]. Both studies also observed an HPV dependent increase in lysine demethylase 6A (KDM6A) or lysine demethylase 6B (KDM6B)—the demethylases that convert H3K27me3 to the di-methylated or mono-methylated forms [28, 30]. Thus, HPV increases expression of the enzymes responsible for creating and removing H3K27me3, complicating our understanding of how the global decrease in H3K27me3 is achieved in an HPV infection.

Mechanistic studies of these HPV dependent changes on epigenetic regulators of gene expression have focused on altered p16 expression, which is present at high levels in nearly all HPV induced tumors [32, 33]. Indeed, p16 was commonly used as a surrogate marker of HPV status during pathological assessment of tumors, but this method to determine HPV status has largely been superseded by the implementation of molecularly based diagnostics [33–35]. p16 and p14 are products of the cyclin-dependent kinase inhibitor 2A (CDKN2A) gene locus and normally function as negative regulators of the cell cycle. p16 induces cell cycle arrest and senescence by inhibiting E2F-mediated transcription. p14 induces cell cycle arrest and apoptosis by facilitating p53 function [36]. Neither of these products inhibit cell cycle progression in HPV induced cancers, as the p53 and Rb pathways have been abrogated by E6 and E7, respectively [20, 21]. However, transcription from the CDKN2A locus is tightly regulated by epigenetic changes, with PRC2 serving a key role in repressing expression by catalyzing tri-methylation of H3K27 across this locus. Subsequently, the polycomb repressive complex 1 (PRC1) is recruited to maintain a transcriptionally repressive chromatin environment that is typically reflected by a local increase in DNA methylation [31]. Thus, this well-characterized locus serves as a good model of epigenetic dysregulation induced by HPV infection [37, 38].

In a detailed series of tissue culture based experiments, evidence was obtained to suggest that the HPV-mediated reduction in H3K27me3 and subsequent transcriptional activation of p16 required E7 to induce expression of the H3K27 specific demethylases KDM6A and KDM6B [28, 39]. Knockdown of either KDM6A or KDM6B in HPV+ cervical carcinoma cell lines not only reduced p16 expression, but also induced cell death [28, 39]. Subsequent experiments showed that E7 expression in cell lines induced an acute dependence on KDM6B for growth. E7 expressing cells were similarly dependent on p16 expression, as treatment with a KDM6 selective small molecule inhibitor was cytotoxic in multiple cervical carcinoma cell lines [28, 39]. Thus, HPV E7 expressing cells are uniquely vulnerable to a targeted agent affecting an epigenetic regulator of cellular gene expression.

While most of this work was done in tissue culture models, similar changes in expression of H3K27me3 regulators were reported in individual cervical carcinomas [28–30]. An analysis of these effects has not been done in any HPV+ oral cavity tumors or on a large scale for any HPV induced tumor site. As such, it remains an open question as to whether these changes in epigenetic regulators are specific for distinct subsites of HPV induced tumors, and how frequently they occur in clinical samples. This information is clearly relevant, considering that cancerous cells expressing E7 appear preferentially sensitive to KDM6 inhibition [28, 39], which could represent a new therapeutic approach for their treatment.

In this study, we used data from over 800 human cervical [40] and head and neck tumors [41] from The Cancer Genome Atlas (TCGA) to determine how high-risk HPV oncogene expression alters expression of H3K27me3 regulators in human tumors. We assessed the impact of HPV status on the transcript levels of key regulators of H3K27 methylation, including EZH2, KDM6A, and KDM6B. We also examined the effect of HPV status on gene expression and DNA methylation status across the CDKN2A genomic region in these two distinct patient cohorts. We found that HPV+ tumors from both anatomical sites exhibited high levels of p16 and p14. Furthermore, HPV+ tumors also expressed higher levels of EZH2 and KDM6A. Analysis of CpG methylation also detected two sites in the CDKN2A locus with consistently altered methylation in HPV+ tumors versus HPV negative (HPV-) samples. Taken together, this data validates conclusions drawn from tissue culture studies and identifies remarkable similarities between the effects of HPV on gene expression and methylation in both cervical and oral tumors in large human cohorts. These results also provide compelling evidence from human tumors that support the current model of HPV dysregulation of H3K27 methylation and cognate gene expression via the upregulation of KDM6A.

## RESULTS

### Impact of HPV status on CDKN2A and CDKN2B expression in human tumors

**Figure 1:**
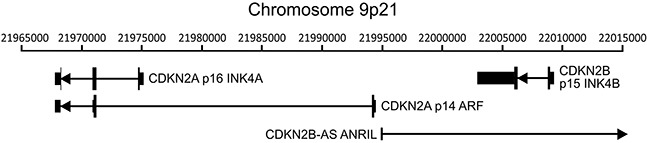
Organization of the CDKN2A and CDKN2B loci The cyclin-dependent kinase inhibitor 2A (CDKN2A) locus encodes two functionally unrelated protein products named p16 INK4A and p14 ARF. The adjacent CDKN2B locus encodes p15 INK4B. This gene cluster also encodes a long non-coding RNA transcribed in the antisense direction named CDKN2B-AS or antisense non-coding RNA in the INK4 locus (ANRIL). The position of transcripts (black lines), exons (black vertical bars), coding regions (thick black vertical bars), and orientation (direction of arrowheads) of the major transcripts in this region of chromosome 9 are indicated.

**Figure 2:**
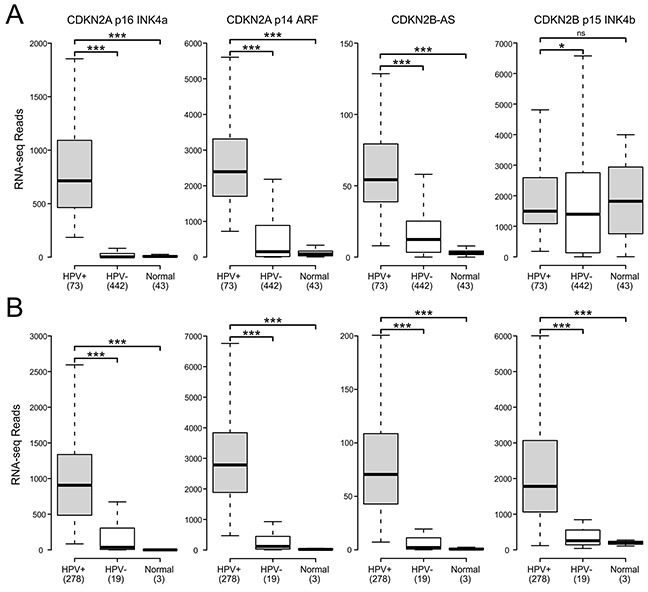
HPV perturbation of expression of the CDKN2A and CDKN2B genes in human head & neck and cervical carcinomas Normalized RNA-Seq data extracted from the TCGA database for the HNSC **(A)** and CESC **(B)** cohorts for HPV+, HPV-, and normal control tissues. Numbers in brackets refer to the number of samples included in each analysis. * p≤0.05, ** p≤0.01, *** p≤0.001, ns- not significant.

The CDKN2A and adjacent cyclin-dependent kinase inhibitor 2B (CDKN2B) loci encode three distinct tumor suppressors: p16 INK4A, p14 ARF, and p15 INK4B. This gene cluster also encodes a long non-coding RNA transcribed in the antisense direction that is referred to as CDKN2B-AS or antisense non-coding RNA in the INK4 locus (ANRIL) (Figure 1). Expression of all three coding genes from this cluster is silenced by polycomb repressive complexes (PRCs) during normal cell growth [31]. While HPV induced cancers commonly express high levels of the p16 product of the CDKN2A locus [32, 33], the expression levels of p14, p15, and the non-coding CDKN2B-AS RNA have not been studied as extensively. We analyzed the TCGA Illumina HiSeq RNA expression data from the head and neck squamous cell carcinoma (HNSC) and cervical carcinoma (CESC) cohorts for expression of all four genes (Figure 2). As expected, HPV+ samples from both the HNSC and CESC cohorts had greatly increased levels of p16 mRNA expression compared to HPV- tumors and normal control tissue. Like p16, a similar upregulation of p14, p15, and CDKN2B-AS was observed in HPV+ cancers with respect to HPV- cancers. These results indicate that PRC-mediated repression is abrogated across the entire CDKN2A and CDKN2B regions of the INK4 locus by HPV infection. The only major difference appears to be the high level of p15 expression present in the normal control samples for the HNSC cohort, which likely reflects some tissue specific, HPV independent regulation.

### Impact of HPV status on expression of PRC2 components and BMI1 in human tumors

**Figure 3:**
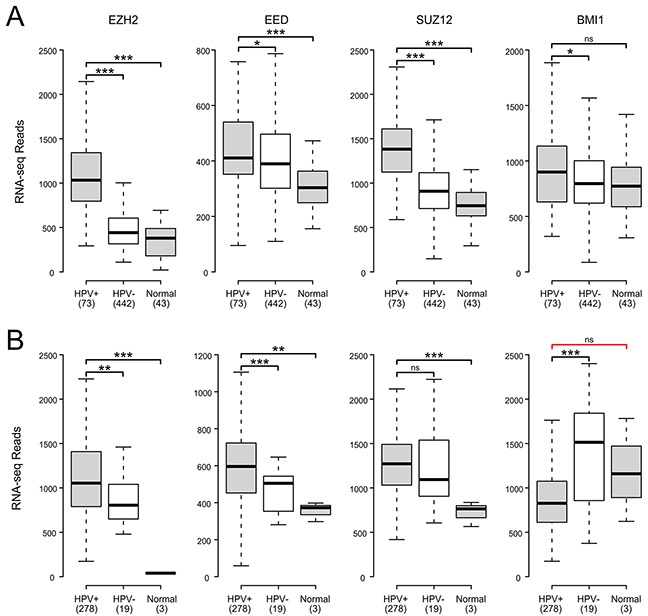
HPV perturbation of expression of polycomb components that may regulate CDKN2A transcription in human head & neck and cervical carcinomas Normalized RNA-Seq data extracted from the TCGA database for the HNSC **(A)** and CESC **(B)** cohorts for HPV+, HPV-, and normal control tissues. Numbers in brackets refer to the number of samples included in each analysis. * p≤0.05, ** p≤0.01, *** p≤0.001, ns - not significant, red bracket indicates a comparison that did not achieve significance with a power value <0.8.

The H3K27 methyltransferase activity of PRC2 relies on the catalytic subunit EZH2, as well as the embryonic ectoderm development (EED) and suppressor of zeste 12 (SUZ12) components. Multiple studies have reported EZH2 upregulation in HPV+ cancer cell lines, with similarly higher expression reported in small studies of HPV+ cervical carcinomas [28–30]. Much less is known about the effect of HPV status on the other PRC2 core components. Analysis of TCGA data reveals significantly elevated expression of all three PRC2 core components compared to normal control tissue in both HNSC and CESC (Figure 3). All but SUZ12 in the CESC cohort are similarly elevated as compared to HPV- cancers. Thus, the core components of the PRC2 methyltransferase are all consistently transcribed at high levels in HPV+ human tumors. This is in stark contrast to the reported decreases in global H3K27me3 [28, 30] and the upregulation of the CDKN2A and CDKN2B loci we observe in HPV+ tumors.

Tri-methylation of H3K27 by PRC2 is proposed to recruit PRC1 via its B lymphoma Mo-MLV insertion region 1 (BMI1) component to maintain a transcriptionally repressive state [27]. A previous study reported that BMI1 expression was reduced by transduction of HPV E6 and E7 into human foreskin keratinocytes (HFKs) [30]. A reduction in BMI1 is consistent with a reduction of repression of the CDKN2A locus by PRC1. BMI1 expression in HPV+ CESC tumors was lower than in HPV- or normal control tissues (Figure 3). However, its expression was generally the same or higher in HPV+ HNSC tumors compared to the HPV- or normal control tissues (Figure 3). The lack of a consistent reduction in BMI1 expression in HPV+ tumors suggest that it is not a key factor in HPV activation of CDKN2A and CDKN2B transcription. This is supported by a smaller independent study that found no correlation between p16 and BMI1 expression in head and neck cancers [42].

### Impact of HPV status on expression of KDM6A and KDM6B in human tumors

**Figure 4:**
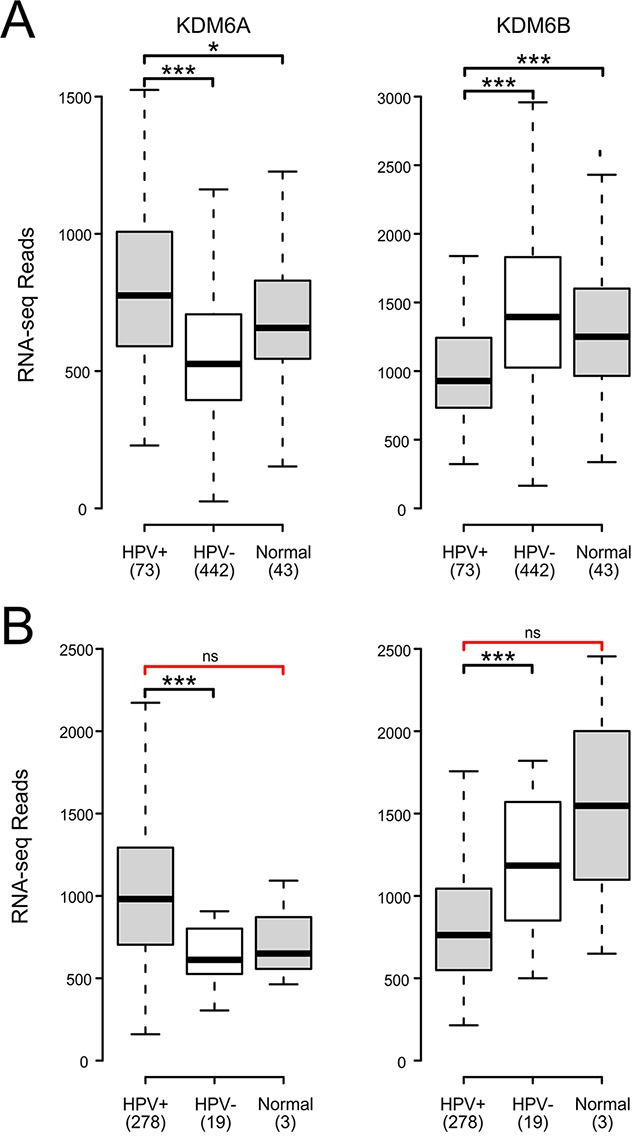
HPV perturbation of expression of the lysine demethylases KDM6A and KDM6B in human head & neck and cervical carcinomas Normalized RNA-Seq data extracted from the TCGA database for the HNSC **(A)** and CESC **(B)** cohorts for HPV+, HPV-, and normal control tissues. Numbers in brackets refer to the number of samples included in each analysis. * p≤0.05, ** p≤0.01, *** p≤0.001, ns- not significant, red brackets indicate a comparison that did not achieve significance with a power value <0.8.

The KDM6A and KDM6B demethylases convert H3K27me3 to the di-methylated or mono-methylated forms [43]. Multiple studies have reported that upregulation of one or both of the KDM6 demethylases occurs in cell lines when HPV E7 is expressed. Furthermore, knockdown of KDM6B or treatment with a small molecule KDM6 inhibitor reduces p16 expression in these models, suggesting a direct causal relationship between KDM6 activity and p16 transcription [28, 39]. Our analysis of the expression of KDM6A reveals that it is significantly upregulated in HPV+ tumors compared to HPV- tumors in both the HNSC and CESC cohorts (Figure 4). In contrast, KDM6B expression is significantly reduced in HPV+ cancers compared to HPV- tumors in both the HNSC and CESC tumor types (Figure 4). Significant changes in KDM6A and KDM6B expression were similarly noted between HPV+ and normal control tissues for the HNSC samples. However, no significant differences were observed for the CESC samples, which could reflect the limited number of normal control samples in this cohort. Overall, the increased expression of KDM6A suggests that it is more likely to play a role in dysregulation of H3K27me3 mediated regulation of transcription in HPV+ cancers.

### Assessment of the DNA methylation status of the CDKN2A and CDKN2B loci in human tumors

**Figure 5:**
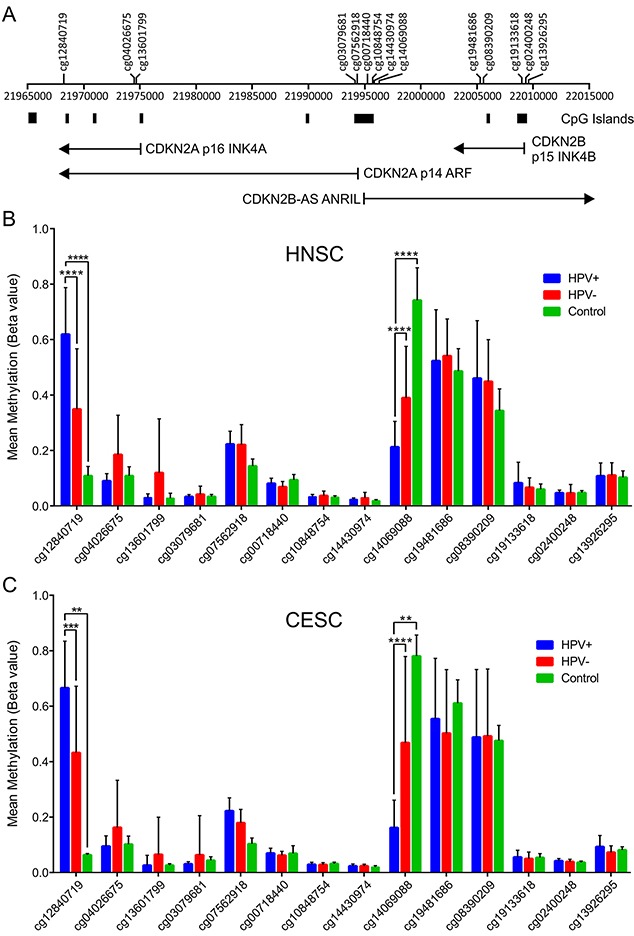
HPV perturbation of DNA methylation at the CDKN2A and CDKN2B loci in human head & neck and cervical carcinomas HPV+ cancers exhibit markedly altered methylation from HPV- tumors or normal control tissues at two regions flanking the CDKN2A locus. **(A)** Location of the transcripts (black lines), CpG islands and methylation probes used in this study. Normalized methylation data was extracted from the TCGA database for the HNSC **(B)** and CESC **(C)** cohorts. The mean methylation levels for HPV+, HPV-, and normal control tissues for all probes located in the vicinity of the CDKN2A and CDKN2B loci are shown. ** p≤ 0.01, *** p≤ 0.001, **** p ≤ 0.0001.

HPV-mediated changes in epigenetic regulation of transcription of the CDKN2A and CDKN2B gene would be expected to be reflected in methylation status of CpG dinucleotide sequences in or adjacent to these loci. Indeed, a previous report identified altered methylation at 4 CpG loci in this genomic region that correlated with HPV status [30, 38]. We analyzed the Infinium HumanMethylation450 BeadChip array data for the TCGA HNSC and CESC cohorts for consistent alterations in DNA methylation in this region of the genome (Figure 5). A region of DNA upstream of the p14 transcript (probe cg14069088; see Figure 5A for location) was consistently identified to be hypomethylated in HPV+ tumors in both the HNSC and CESC cohorts compared to HPV- tumors or normal control tissue. No differences in methylation were detected in the remainder of the probes across the transcribed regions of these loci in HPV+ versus HPV- or normal control samples. In contrast, a hypermethylated region of DNA was identified within the 3′-end of the CDKN2A locus (probe cg12840719; see Figure 5A for location) in HPV+ tumors regardless of anatomical location. Thus, altered methylation is detected at the CDKN2A locus in HPV+ tumors, which is consistent with an epigenetic mechanism for transcriptional activation of this locus by HPV infection.

## DISCUSSION

HPV infection and subsequent expression of the E6 and E7 oncogenes leads to a large-scale reprogramming of gene expression. This is likely further exacerbated in high-risk HPV+ tumor cells, which typically express higher levels of these powerful viral regulatory proteins compared to levels found in the context of normal infections. Consequently, high-risk HPV transformed cells exhibit many changes in gene expression that contribute to the acquisition and maintenance of cancerous phenotypes unique to viral transformation [13]. In particular, dysregulation of H3K27me3 homeostasis by the E7 proteins from high-risk HPVs contributes to increased expression of loci normally repressed by PRCs, including the CDKN2A locus encoding p16 INK4A [26, 28, 39]. Interestingly, while strong p16 expression is a near universal hallmark of high-risk HPV+ carcinomas of the cervix or oral cavity [32–34], p16 is not expressed in high-risk HPV+ low-grade lesions and variably expressed in higher grade precancerous lesions [44, 45] or lesions caused by low-risk HPV types [32, 46, 47]. Thus, expression of high-risk HPV oncogenes alone is not sufficient to activate high levels of p16 expression, and additional adaptations acquired during tumor development likely contribute to this process.

While cell culture models have allowed detailed analyses of factors involved in virus mediated activation of p16 gene expression from the CDKN2A locus, far less is known about how similar these effects are to actual HPV+ tumors. In this study, our goal was to determine if the changes in H3K27me3 regulators and their target genes observed in cell culture models of HPV transformation are recapitulated in primary HPV+ tumors from different anatomical locations.

Using data from over 800 primary human tumors, we provide evidence that HPV+ tumors from both the head and neck and cervical sites exhibit remarkably similar alterations in expression of the genes encoded by the CDKN2A locus (Figure 2). It is well established that HPV induces expression of p16 INK4A in both oropharyngeal and cervical carcinomas [33, 34]. While overexpression of the p14 ARF gene from this locus has been observed in these cancers [37, 38], this has not been extensively documented. Our observation that p14 expression, like that of p16, is similarly elevated in comparison to HPV- tumors or normal control tissue clearly demonstrates that HPV activates expression of the entire CDKN2A locus. Furthermore, this activation extends into the adjacent CDKN2B locus, which is also regulated by PRCs [31]. Indeed, both the long non-coding RNA, CDKN2B-AS ANRIL, and p15 INK4B are induced in HPV+ cervical carcinomas with respect to HPV- tumors and normal control tissue; similarly, high levels of both CDKN2B encoded genes are expressed in HPV+ head and neck cancers (Figure 2). Existing literature from independent cohorts confirms that p15 is expressed at lower levels in normal cervical tissue compared to cervical carcinomas [48, 49], supporting our conclusions from the TCGA CESC data. In addition, an independent analysis of a subset of the TCGA HNSC cohort used in our analysis observed similarly high, albeit variable, levels of p15 in HPV+ and normal control samples [50]. Taken together, the similarities between the normalized expression of all four gene products from the CDKN2A and CDKN2B gene loci in HPV+ tumors from both the cervix and oropharynx is extraordinary. These results clearly demonstrate the dominant nature of viral reprogramming of gene expression in these cancers with respect to normal control tissue and HPV- cancers.

A simple mechanism to explain the observed increase of CDKN2A and CDKN2B expression in HPV+ cells would be a decreased expression of the proteins necessary for the H3K27 methyltransferase activity of the PRC2. In tissue culture models, expression of the EZH2 methyltransferase is increased by the presence of HPV oncogenes, and no significant change is observed for the EED and SUZ12 core proteins also required for catalytic activity [28, 30]. Similarly, in HPV+ tumors from both anatomical sites, we find that these genes are expressed at levels equivalent or even higher than normal control tissue and HPV- samples (Figure 3). Furthermore, an analysis of ten biopsies of normal cervical tissue, HPV+ high-grade precancerous lesions, and HPV+ cervical carcinomas detected similarly elevated levels of EZH2 protein in the HPV expressing samples [29]. Thus, there is a remarkably close agreement between the finding reported from tissue culture models and what is observed in human tumors, confirming that a reduction in the overall level of PRC2 expression is not likely responsible for CDKN2A and CDKN2B activation.

Silencing of euchromatin by PRC2 is stabilized and maintained by PRC1. Cell culture experiments suggest that expression of BMI1, a core component of PRC1, is reduced in E6/E7 transduced HFKs [30] and BMI1 expression is inversely correlated with HPV titer in cervical carcinomas [51]. Our analysis of primary human cervical carcinomas indicates that BMI1 expression is significantly reduced in HPV+ tumors with respect to HPV- tumors. However, this is not the case in head and neck tumors, where it is modestly upregulated (Figure 3). For this reason, it seems unlikely that the induction of CDKN2A and CDKN2B expression observed in both these cancers is related to a reduction in expression of the BMI1 component of PRC1. This is further supported by a study reporting that BMI1 protein expression was lost in less than half of the 40 p16 positive oropharyngeal carcinoma oral tumors tested [42]. Thus, a loss of BMI1 expression appears unrelated to increased p16 transcription, at least in the context of oropharyngeal tumors.

An alternative method of activating the CDKN2A and CDKN2B loci could be via the removal of the repressive H3K27me3 modification catalyzed by the PRC2 complex. This histone modification is erased by the KDM6A and KDM6B demethylases. Previous work in tissue culture models established that transduction of HPV E7 into HFKs induced KDM6A and/or KDM6B mRNA and protein levels [28, 30]. Mechanistically, induction of KDM6B was independent of the interaction of E7 with Rb. More importantly, siRNA knockdown of KDM6B antagonized p16 induction by E7, suggesting that upregulation of these demethylases is the mechanism by which HPV reduces global H3K27me3 levels and activates p16 expression [28, 39]. Our analysis of primary human tumors indicates that KDM6A expression is significantly increased in HPV+ head and neck and cervical carcinoma cells with respect to HPV- tumors and likely normal control tissue. However, this is not the case for KDM6B, which is significantly reduced (Figure 4). These data fully support the model proposed from tissue culture experiments, in which enhanced expression of KDM6A, but not KDM6B, by HPV is related to the dysregulation of H3K27me3 homeostasis and subsequent activation of CDKN2A and likely CDKN2B transcription in both these cancer types. One small study reported that expression of both KDM6A and KDM6B is substantially reduced in HPV+ mildly dysplastic lesions; however, stepwise increases were observed for both demethylases in precancerous lesions and cervical carcinoma, such that the carcinomas express both demethylases at levels similar to normal control tissues [52]. This pattern of re-expression correlates well with the acquisition of p16 expression observed during cervical carcinoma progression.

Accompanying the global reduction in H3K27me3, HPV+ head and neck cancers and cervical carcinomas exhibit numerous alterations in DNA methylation [53, 54]. Indeed, we recently reported a methylation signature that predicts HPV status in HNSC samples [55]. Our analysis of methylation data for all the probes across the CDKN2A and CDKN2B loci for the TCGA HNSC and CESC cohorts revealed that there were two consistent alterations in DNA methylation relative to methylation levels present in HPV- tumors and normal tissue (Figure 5). A hypomethylated region of DNA was identified on the south shore of the CpG island located at the start of the p14 transcript in HPV+ tumors in both the HNSC and CESC cohorts. Genome-wide studies have clearly shown that methylation in the immediate vicinity of the transcription start site blocks initiation [56]. As such, the greatly reduced methylation in HPV+ samples at this region is fully consistent with enhanced CDKN2A expression. In addition, we also observed a hypermethylated region of DNA on the north shore of the CpG island closest to the third exon of the p14 and p16 transcripts. Hypermethylation at this location has been described in other studies of HPV+ head and neck and cervical carcinomas using alternative probes [38, 57, 58]. These studies have established that p16 and p14 expression in a given tumor sample is strongly correlated with increased methylation at this position in the CDKN2A locus [38, 57]. Hypermethylation of gene-bodies has recently been shown to occur during elongation through a mechanism whereby RNA Polymerase II recruits Su(var)3-9, Enhancer-of-zeste and Trithorax domain containing 2 (SETD2), a histone methyltransferase, which then tri-methylates H3K36. DNA Methyltransferase 3 (DNMT3) recognizes and binds H3K36me3, leading to the methylation of CpGs [59, 60]. Thus, along with promoter hypomethylation, gene-body methylation correlates well with the observed enhanced transcription from the CDKN2A locus, providing further support for an epigenetic mechanism as the basis for HPV induction of expression from this locus.

Taken together, our analysis of over 350 HPV+ head and neck or cervical carcinomas in comparison with over 450 HPV- cancers from their respective anatomical sites demonstrates that HPV consistently activates expression of both the CDKN2A and CDKN2B loci in these cancers. Interestingly, this extends beyond just p16, with most tumors expressing the other gene products from these loci including p14, ANRIL, and p15. In these cancers, HPV oncogene expression similarly dysregulates expression of key regulators of H3K27me3, including members of PRC2 and the demethylases KDM6A and KDM6B. As a likely result of these and other alterations in epigenetic regulation, the levels of methylation in the center of the CDKN2A and CDKN2B loci are significantly decreased in cells expressing high-risk HPV oncogenes.

Importantly, epigenetic reprogramming of gene expression induced by HPV should be reversible, and could represent a unique therapeutic target. Paradoxically, cervical carcinoma cell lines or other tumor cell lines expressing HPV E7 from several high-risk HPVs appear to require continuous p16 expression for survival. Although p16 normally serves as a tumor suppressor, for as yet unknown reasons, it becomes necessary for continued growth in cells expressing HPV E7. As HPV+ cells require KDM6 demethylase activity to maintain p16 expression, they are sensitive to small molecule inhibitors of KDM6 in cell culture models [28, 39]. However, it has not been determined if actual primary HPV+ head and neck or cervical carcinoma cells will respond similarly. Our analysis of the epigenetic regulators that control p16 expression finds many commonalities between the hundreds of tumors making up the TCGA CESC and HNSC cohorts and cell culture models, strongly suggesting that testing of KDM6 inhibitors should be aggressively pursued as a novel therapy of HPV+ cancers.

## MATERIALS AND METHODS

### RNA expression comparisons and statistical analysis

Level 3 RSEM normalized Illumina HiSeq RNA expression data for the TCGA head and neck cancer (HNSC) and cervical carcinoma (CESC) cohorts was downloaded from the Broad Genome Data Analysis Centers Firehose server (https://gdac.broadinstitute.org/). Although rare HPV+ samples have been identified in the TCGA bladder urothelial carcinoma (BLCA) and colon adenocarcinoma (COAD) cohorts, they were not present in sufficient numbers for any useful comparisons. For all genes except p16 and p14, the gene level Firehose dataset was used. For p16 and p14, the gene isoform level Firehose dataset was used (uc003zpk.2 and uc003zpl.2 respectively) to adequately discriminate between these two different products of the CDKN2A gene. Normalized expression data was extracted into Microsoft Excel and the HPV status was manually curated based on published datasets [40, 41, 61, 62]. For each gene analyzed, primary patient samples with known HPV status were grouped as HPV+, HPV-, or normal control tissue. Patient samples with unknown HPV status were omitted from our calculations, as were samples obtained from secondary metastatic lesions. This resulted in 73 HPV+, 442 HPV-, and 43 normal control samples with data available for the HNSC gene expression analysis and 278 HPV+, 19 HPV-, and 3 normal control samples available for the cervical carcinoma gene expression analysis. Boxplot comparison of gene expression was performed using BoxPlotR (http://shiny.chemgrid.org/boxplotr/) and assembled into final form using CorelDRAW. For the box plots, center lines show the medians, box limits indicate the 25th and 75th percentiles as determined by R software and whiskers extend 1.5 times the interquartile range from the 25th and 75th percentiles. Statistical significance was calculated using Graphpad Prism v6.01. p-values were assigned using a one-tailed non-parametric Mann-Whitney U test. Post-hoc power calculations were performed with G*Power software version 3.1.9.2 [63], using post-hoc t-test family calculations, with effect size selected as 0.8 and α = 0.05. All comparisons achieved a power value >0.8, or demonstrated significant differences, unless otherwise noted in the text.

### DNA methylation comparisons and statistical analysis

Level 3 Infinium HumanMethylation450 BeadChip array data for the TCGA HNSC and CESC cohorts was downloaded from the Broad Genome Data Analysis Centers Firehose server (https://gdac.broadinstitute.org/). 14 methylation probes were identified to be present in the CDKN2A and adjacent CDKN2B region: cg00718440, cg02400248, cg03079681, cg04026675, cg07562918, cg08390209, cg10848754, cg12840719, cg13601799, cg13926295, cg14069088, cg14430974, cg19133618, cg19481686. Methylation data for all patient samples for these probes were extracted from the Firehose files and assembled into a table using Microsoft Excel. HPV status was manually curated based on published datasets [40, 41, 61, 62]. For each gene analyzed, primary patient samples with known HPV status were grouped as HPV+, HPV-, or normal control tissue. Patient samples with unknown HPV status were omitted from our calculations, as were samples obtained from secondary metastatic lesions. This resulted in 73 HPV+ tumors, 442 HPV- tumors, and 43 normal control samples with data available for the HNSC methylation analysis and 278, 19, and 3 samples respectively available for the CESC methylation analysis. The average methylation Beta-value and standard deviation was calculated for each probe for each sample type in each cohort. Data was plotted using Excel and assembled into final form using CorelDRAW. For statistical analysis, Beta-values were converted to M-values using the following equation to improve homoscedasticity of the data:

$$M_{i}=\log_{2}(\frac{Beta_{i}}{1-Beta_{i}})$$

where M_i_ and Beta_i_ are the M-value and Beta-value of the i^th^ interrogated CpG site [64]. The data was analyzed using Graphpad Prism v6.01. p-values were assigned using non-parametric Kruskal-Wallis test and Dunn's multiple comparisons test.
